# Supplementary material for: Qualitative study investigating the perceptions of parents of children who failed vision screening at the age of 4–5 years
Source: BMJ Paediatr Open. 2018 Sep 14;2(1):e000307. doi: 10.1136/bmjpo-2018-000307 (PMC6144893; doi:10.1136/bmjpo-2018-000307)
Supplement: Author's manuscript [file bmjpo-2018-000307.draft_revisions.pdf]

BMJ Paediatrics Open

BMJ Paediatrics Open is committed to open peer review. As part of this commitment we make the peer review history of every article we publish publicly available.

When an article is published we post the peer reviewers' comments and the authors' responses online. We also post the versions of the paper that were used during peer review. These are the versions that the peer review comments apply to.

The versions of the paper that follow are the versions that were submitted during the peer review process. They are not the versions of record or the final published versions. They should not be cited or distributed as the published version of this manuscript.

BMJ Paediatrics Open is an open access journal and the full, final, typeset and author-corrected version of record of the manuscript is available on our site with no access controls, subscription charges or pay-per-view fees (http://bmjpaedsopen.bmj.com).

If you have any questions on BMJ Paediatrics Open's open peer review process please email info.bmjpo@bmj.com

BMJ Paediatrics Open

Too young for spectacles? Understanding children's eye-care: A qualitative study

Journal:	*BMJ Paediatrics Open*
Manuscript ID	bmjpo-2018-000307
Article Type:	Original article
Date Submitted by the Author:	30-Apr-2018
Complete List of Authors:	Bruce, Alison; Bradford Institute for Health Research, ; University of York, Health Sciences<br>Sanders, Tom; University of Sheffield, School of Health and Related Research (ScHARR)<br>Sheldon, Trevor; University of York, Department of Health Sciences
Keywords:	Comm Child Health, Ophthalmology, Patient perspective, Qualitative research, Screening

SCHOLARONE™
Manuscripts

Confidential: For Review Only

Too young for spectacles? Understanding children's eye-care: A qualitative study

Alison Bruce (corresponding author), Post-doctoral Research Fellow and Head Orthoptist
Bradford Institute for Health Research, Bradford Teaching Hospitals NHS Trust, Duckworth Lane, Bradford, BD9 6RJ
01274 383414
alison.bruce@bthft.nhs.uk

Tom Sanders, Acting Director of Postgraduate Research
School of Health and Related Research (ScHARR), Section of Public Health, University of Sheffield, Regent Court, 30 Regent Street, Sheffield, S1 4DA
0114 222 4293
tom.sanders@sheffield.ac.uk

Trevor A Sheldon, Professor of Health Services Research and Policy
Department of Health Sciences, University of York, Heslington, York, YO10 5DD
01904 321521
Trevor.Sheldon@york.ac.uk
01904 321521

Word count: 2490

Confidential: For Review Only

Abstract

Objective: To explore reasons for non-attendance following referral from childhood vision screening and non-adherence to prescribed spectacle wear.

Design: Semi-structured interviews were conducted with parents of children who had failed vision screening at age 4-5 years. Four were parents of children who never attended follow-up, 11 had children who attended but did not adhere to spectacle wear and 5 parents of children who had attended and adhered. Interviews were recorded and transcribed verbatim, thematic analysis based on the constant comparative method was undertaken.

Results: Parents beliefs led to uncertainty about the benefit of treatment, with parents testing their children to confirm the presence of a vision deficit and seeking advice from other family and community members. The stigma of spectacle wear explained the resistance of some to their child's treatment with the maintenance of 'normality' often more important than clinical advice. The combination of parents own health beliefs, stigma and the practicalities of attending appointments together influenced parental decisions. Attendance following vision screening and the decision to adhere to spectacle wear were primarily based on the perceived severity of the visual reduction with the perceived benefit of spectacle wear outweighing any negative consequences.

Conclusions: Health care professionals require a greater understanding of parents' decision making processes in order to provide personalised information. Knowledge of the cues to attendance and adherence provides policy makers a framework with which to review the barriers, develop strategies and redesign children's eye care pathways.

Confidential: For Review Only

INTRODUCTION

Decreased vision in young children is commonly due to the presence of refractive error, strabismus, and/or amblyopia.[1] The presence of one or more of these conditions can result in a visual deficit in the developing infant. Most young children with refractive error or amblyopia do not however demonstrate obvious signs or symptoms, making it difficult for parents to identify, and vision screening is recommended in the United Kingdom at age 4-5 years.[2] Early detection programmes can only be effective if those identified with poor vision are appropriately treated. Around 30% fail to attend follow up[3] and studies have shown poor adherence to spectacle wear,[4-6] therefore a high proportion of children with identified needs do not access ophthalmic services and subsequent treatment.

Socioeconomic factors have been reported to influence access to children's eye care[7] and adherence with spectacle wear.[8] In USA difficulty navigating the health care system, cost and loss and breakage of spectacles has been found to prevent attendance and adherence leading to inequalities in visual health.[9] Factors such as cultural sensitivity and health beliefs have been associated with differences in access, uptake and adherence decisions in adults.[10] People's perception of the risk of certain health problems also influences healthcare utilisation.[11] Current literature therefore indicates a number of potential influences impacting on access and uptake of children's eye services. The aim of this study is to explore in depth parents' experiences and understanding of their children's eye care in order to better comprehend why there is relatively low uptake of services and variable adherence to treatment.

METHODS

The population based vision screening programme in Bradford, a large multi-ethnic city in England with high levels of deprivation, is offered to children in their year of school entry, aged 4-5 years. Children failing to achieve the pass criterion set by the UK National Screening Committee (≤0.20 logMAR in both eyes) are referred for examination, either to a

https://mc.manuscriptcentral.com/bmjpo

Confidential: For Review Only

community optometrist or the hospital eye service (HES) and subsequently followed-up in order to measure the visual acuity with prescribed spectacles. As part of a research programme[12] children were followed-up for two years following vision screening to explore their vision and early literacy. Children were defined as adherent (wearing prescribed spectacles at follow-up assessments) or not. Parents of children were purposively selected to participate in this study if their child had failed vision screening and either had not attended follow-up appointments at their local optometrist or the Hospital Eye Service (HES) (n=4) or if their child had been observed to be non-adherent to spectacle wear at the follow-up assessments (n=11). For comparison puposes, parents of four children identified as adhering with spectacle wear were also invited to participate. Twenty parents of 19 children in five schools consented to participate, four parents required interpreters (3 Urdu and 1 Parsi). Semi-structured qualitative interviews were conducted by one member of the study team (AB) in the child's school at a time suitable for the parent, two mothers in one school arrived at the same time and a combined interview was undertaken. Both the mother and father of one child were interviewed together; the father was the main contributor therefore only his discourse is included in the analysis.

A topic guide based on Health Belief theory[13] was used to ensure the key areas of interest were explored in depth (Table 1).[14] The Health Belief model consists of six key constructs (Table 1) which together provide an explanatory framework for the adoption of preventative health behaviour.[15] Parent's views of the likelihood of their child having a vision problem, the perceived severity, along with perceived barriers and benefits of treatment were elicited. In particular their views on vision and spectacle wear in children and the influence of their own experiences and those of family members were explored. Interviews were audio recorded and transcribed verbatim, in addition, the non-English transcripts were listened to by a bilingual research assistant to confirm accuracy of the interpretation. Two members of the study team (AB and TS) reviewed the transcripts independently and then together developed a thematic analysis identifying key themes and concepts (Table 2).[14,16] Written consent was

Confidential: For Review Only

obtained prior to each interview. Ethics approval was obtained from the National Research Ethics Committee Yorkshire and the Humber- South Yorkshire UK (Ref 13/YH/0379).

RESULTS

In-depth interviews were completed with 17 mothers and 3 fathers; 14 parents were South Asian, 5 were white British and 1 African heritage (Table 2). Our analysis identified themes which highlighted pragmatic reasons for non-attendance and non-adherence and also those related to health beliefs about illness, disease and consultation behaviour, illustrative quotes are presented both in the text and in Table 3.

Non-attendance and non-adherence

Parents whose child had never attended follow-up demonstrated resistance to being told their child had a vision problem and rationalised the non-attendance.

They'd given me a letter to take to the opticians, but I didn't ever think nought from it, I just thought I dunno…

Yeah but it's not my fault that like hers are like that, obviously she's born with eyes like that (mother 11).

Parents of children who had never attended reported their child's fear of the eye examination; this was not reported by parents whose children did actually attend.

…they said to go and have him checked up but he's been scared (mother 17).

Attended and non-adherent

Parents who attended reported a series of practical obstacles ranging from transport problems to difficulties supporting treatment which they had to overcome. The following

Confidential: For Review Only

parent struggled to attend appointments and in addition the child's school expressed concern about their absence, all of this added to the inconvenience.

> **Awkward, really awkward because it's generally in school time and she doesn't like missing any time at school…. (mother 6).**

Parents found it difficult to encourage their children to wear spectacles and some had enlisted the support of the child's school.

So (erm), many times many occasions I've forgot glasses at home and then I gave one pair to school as well to resolve this issue (mother 12).

Parents with limited English language skills relied on family members to assist with transportation or the interpretation of clinical findings, in some cases this led to a lack of understanding of the underlying problem.

> **If I knew that she had one of those wavering eyes then I would definitely have taken her but I didn't understand that from the letter (mother 14).**

Parents carried out their own assessments suggesting professional opinion did not convince them of the benefits of glasses. Conversely their support or rejection of glasses wear was based on their own observations or those of significant others.

> **….my wife was saying "she can see alright, she doesn't need to wear glasses." (father 2)**

Confidential: For Review Only

When there was doubt regarding the child's ability to perform the vision test the uncertainty affected parental reinforcement of spectacle wear.

> **…. he's got very short attention span so he wasn't interested so he just liked to look around so I don't know giving up and the like. So I was a bit concerned whether the eyesight was actually you know was a problem… (mother 8).**

Some parents were concerned that spectacles were required at such a young age, and expressed doubt that the child had a genuine problem.

….oh no she's too young so I thought she's too young and (erm) why she's going to wear glasses (mother 12).

Other parents claimed that they were not told how much improvement there had been, affecting their ability to monitor progress and reinforce the treatment.

> **It would be good to be updated on whether her eyesight was getting better or worse (mother 2).**

A number of parents claimed stigma experienced by children from wearing spectacles or eye patches played a part in non-adherence, and was not always recognised by the vision specialists. So parents, in an effort to reduce negative social impact, were inclined to restrict wearing spectacles or eye patches to the home.

> **…but she didn't want the stigma of being bullied and being called pirate and things like that so I said she could wear them at home instead (mother 6).**

Confidential: For Review Only

Parental resistance to spectacle wear was partly due to the belief that they were a barrier to normality and fitting in. Mother 7 (Table 3) relayed a narrative that her child did not need spectacles as she coped well without them, perhaps to offset the stigmatisation from peers. Labels such as 'clever' or 'intelligent' to negatively describe spectacle wear were also given in some cultures.

> **…in Zimbabwe people who wear glasses they are seen as people who are really well educated. They say, "You're wearing glasses, you think you're so.. educated." (mother 4).**

Attended and adhered

Parents whose child both attended and adhered to treatment gave examples of the perceived severity of the vision loss and the benefits of spectacle wear were only reported by parents whose children were adherent (Table 3).

> **….if a kid cannot see properly and keeps getting headaches because you know he's trying to concentrate, it's going to affect him in his education, it's going to affect him when he's playing and during life as well, so it's very important (father 3).**

Confidential: For Review Only

DISCUSSION

Deep rooted health beliefs played an important part in explaining attitudes towards vision care in children starting primary school. The view that children 'should not' require spectacles and other cultural health beliefs gave rise to uncertainty surrounding the potential benefits of spectacle wear, viewed by some as unnecessary. In these cases advice sought from family or community members was more influential, particularly when it matched parents' personal beliefs. The decision to attend following vision screening and to adhere to spectacle wear was primarily based on the perceived severity of the visual reduction with the benefit of spectacle wear outweighing any negative consequences. Parental observations played an important role in validating the professional assessment, when parental perception did not match clinical opinion adherence was less likely.[17] Parental knowledge tended to dominate decisions where there was discordance between professional and lay knowledge[18] with parents having difficulty accepting clinical advice, particularly when their child did not appear to require spectacles for reading and writing. Concordance around treatment should involve discussion between clinician and parent through which an informed decision regarding treatment can take place.[19] Non-adherence was more likely when shared decision making did not appear to be encouraged and parents disregarded clinical advice. This has been reported in general practice with patients less likely to take medications if their own concerns are not addressed.[18]

Parents whose children had failed vision screening and had not taken their child for further assessment were keen to justify their actions by normalising the eye condition[17,20] or by attributing their decision to another reason, such as the child's fear. These parents did not accept that their child was susceptible to a vision problem and hence appeared not to take responsibility for their child's non-attendance. No support was reported either from family members or from the child's school and this lack of support may also have contributed to non-attendance.

https://mc.manuscriptcentral.com/bmjpo

Confidential: For Review Only

Parents whose children had attended but not adhered to the prescribed treatment based their decisions on a combination of factors including the susceptibility and severity of the condition along with the magnitude of the barriers. The reduction of vision in children this young is not generally associated with overt signs and symptoms and parents found it difficult to accept their child had a vision problem. Parents either reasoned that the test was not age appropriate and their child was too young to perform the test or attributed lack of concentration to behavioural rather than vision problems. It has been reported that limited experience of a health condition affects judgement of its severity, this may also influence parents reasoning to support spectacle wear in their children.[21]

Stigma played a major part in explaining parents' resistance to clinical advice.[22] Appearing 'normal' seemed to outweigh potential benefits[23] and was a strong driver for maintaining a socially acceptable image of the family within the community;[20] a child with spectacles seemed to threaten this image suggesting that the eye condition was not the sole factor impacting on adherence.[24]

Parents who did not consider their child's vision to be impaired and who described barriers such as organising transport or social stigma were less likely to follow recommendations and deemed treatment unnecessary. Where support was available either from family members or from the child's school this provided a cue to action prompting parents to take the first step in attending initial appointments. Several parents stated that school should take a more active role in both supporting attendance and adherence. Some parents lacked confidence in the results of the vision assessment or felt advice was lacking, all these factors added to parents' likelihood of non-adherence to treatment.

Parents with limited English language ability relied on family members to access care and interpret clinical advice, this use of the family network is reported to be unreliable, leading to misinformation and may impact on the children's care.[11] The practical considerations of attending regular appointments particularly in cases of uncertainty about the benefit of

Confidential: For Review Only

treatment therefore posed a dilemma, contributing to and perhaps being the tipping point for non-attendance and non-adherence.

These findings provide insight into the reasons parents either rejected or resisted therapeutic advice, not solely reflecting levels of knowledge,[24] but reflecting an active evaluation of the potential severity of the vision loss and the perceived loss of normality and social stigma[20] compared to the benefit of the treatment.[23]

Our study has limitations, the participants all reside in an area of relative deprivation and their behaviour may not reflect that of parents in more prosperous areas.[25] In addition we did not examine health literacy which may have impacted on the decision making process.[26] The interviews were undertaken in schools and parents may have felt pressurised to be “good” parents. However they demonstrated an affiliation with their child’s school, with no parent choosing to be interviewed in their own home.

Pragmatic strategies are required to improve attendance and adherence. The desire for condition specific information is underestimated by clinicians[27] and provision of personalised information, following vision screening, highlighting the benefits of treatment in young children could improve attendance. The current national development of information leaflets specifically for parents may influence attendance following vision screening.[28] Informed shared decision making is required to promote concordance;[29] parents are less likely to support the treatment if their beliefs are in conflict and their concerns unaddressed.[30] Further study is required into how eye care professionals communicate with parents, and how they present information[30] to ensure the parents can make informed decisions regarding adherence. Stigma was commonly cited by parents as a barrier to adherence.[31] Strategies both at the individual and community level are required with the role of schools as facilitators in reducing stigma and promoting adherence, an area highlighted by parents, requiring future study. Our results illustrate the complexity around attendance and adherence patterns and provide a greater understanding which can inform the redesign of children’s eye care pathways.

Confidential: For Review Only

Competing interests: None declared

Grants: AB is funded by a National Institute for Health Research Post-Doctoral Fellowship Award (PDF-2013-06-050). The views expressed are those of the author(s) and not necessarily those of the NHS, the NIHR or the Department of Health.

Confidential: For Review Only

“What is already known on this topic”

- Reduced vision in young children is commonly related to refractive error, strabismus or amblyopia.
- Following identification of reduced vision at vision screening attendance for eye care appointments is poor with 30% of children failing to attend.
- Treatment consists of spectacle wear and may be combined with wearing an eye patch but adherence to treatment is inconsistent.

“What this study adds”

- Reasons for non-attendance and non-adherence are complex with both pragmatic factors and health beliefs interlinked in parental decision making.
- The decision to adhere to spectacle wear was based primarily on the perceived severity of the visual reduction with the perceived benefit outweighing any negative consequences.
- The barriers and facilitators for attending and adhering to children’s eye care are presented providing information by which interventions to promote spectacle wear can be developed.

Confidential: For Review Only

REFERENCES

1. Robaei D, Rose K, Ojaimi E, *et al.* Visual Acuity and the Causes of Visual Loss in a Population-Based Sample of 6-Year-Old Australian Children. *Ophthalmology* 2005;112(7):1275-82.

2. National Screening Committee. The UK NSC policy on Vision defects screening in children 2013. Available: http://www.screening.nhs.uk/vision-child (Accessed 19/12/2017).

3. Bruce A, Outhwaite L. Uptake, referral and attendance: results from an inner city school based vision screening programme. *Br Ir Orthopt J* 2013;10:41 - 45.

4. Aldebasi YH. A descriptive study on compliance of spectacle-wear in children of primary schools at Qassim Province, Saudi Arabia. *Int J Health Sci (Qassim)* 2013;7(3):291-99.

5. Sharma A, Congdon N, Patel M, *et al.* School-based approaches to the correction of refractive error in children. *Surv Ophthalmol* 2012;57(3):272-83.

6. Maconachie GE, Farooq S, Bush G, *et al.* Association between adherence to glasses wearing during amblyopia treatment and improvement in visual acuity. *JAMA Ophthalmol* 2016;134(12):1347-53.

7. Williams C, Northstone K, Howard M, *et al.* Prevalence and risk factors for common vision problems in children: data from the ALSPAC study. *Br J Ophthalmol* 2008;92(7):959-64.

8. Ma X, Zhou Z, Yi H, *et al.* Effect of providing free glasses on children's educational outcomes in China: cluster randomized controlled trial. *BMJ* 2014;349.

9. Messer DH, Mitchell GL, Twelker JD, *et al.* Spectacle Wear in Children Given Spectacles Through a School-Based Program. *Optom Vis Sci* 2012;89(1):19-26.

10. King's Fund Briefing *Access to health care and minority ethnic groups.* Februay 2006. Available:

https://www.kingsfund.org.uk/sites/default/files/field/field_publication_file/access-to-health-care-minority-ethnic-groups-briefing-kings-fund-february-2006.pdf (accessed 20/12/2017).

11. Dixon-Woods M, Kirk M, Agarwal S, *et al.* Vulnerable Groups and Access to Health Care:A Critical Interpretive Review: National Co-ordinating Centre for NHS Service Delivery

Confidential: For Review Only

and Organisation London: 2005. Available online at http://www.menshealthforum.org.uk/uploaded_files/SDOvulnerablegroups2005.pdf [Accessed 09 November 2017].

12. Bruce A, Fairley L, Chambers B*, et al.* Impact of visual acuity on developing literacy at age 4-5 years: a cohort-nested cross-sectional study. *BMJ Open* 2016;6(2):e010434.

13. Rosenstock IM, Strecher VJ, Becker MH. Social learning theory and the Health Belief Model. *Health Educ Q* 1988;15(2):175-83.

14. Pope C, Ziebland S, Mays N. Analysing qualitative data. *BMJ* 2000;320(7227):114-16.

15. Griffin MJ. Health belief model, social support, and intention to screen for colorectal cancer in older African American men. *Health Promotion & Education* 2012.;51(1):12-22.

16. Glaser G, Stauss, A. *The discovery of grounded theory. Chicago: Aldine,*, 1967.

17. McLachlan S, Mansell G, Sanders T*, et al.* Symptom perceptions and help-seeking behaviour prior to lung and colorectal cancer diagnoses: a qualitative study. *Fam Pract* 2015;32(5):568-77.

18. Britten N, Stevenson FA, Barry CA*, et al.* Misunderstandings in prescribing decisions in general practice: qualitative study. *BMJ* 2000;320(7233):484-88.

19. Jordan JL, Ellis SJ, Chambers R. Defining shared decision making and concordance: are they one and the same? *Postgrad Med J* 2002;78(921):383-84.

20. Prout A, Hayes L, Gelder L. Medicines and the maintenance of ordinariness in the household management of childhood asthma. *Sociol Health & Illn* 1999;21(2):137-62.

21. Bond L, Nolan T. Making sense of perceptions of risk of diseases and vaccinations: a qualitative study combining models of health beliefs, decision-making and risk perception. *BMC public health* 2011;11(1):943.

22. Goffman E. *Stigma: Notes on the Management of Spoiled Identity*. Englewood Cliffs, N.J: Prentice-Hall, Inc, 1963.

23. Dixon-Woods M, Awan M, Gottlob I. Why is compliance with occlusion therapy for amblyopia so hard? A qualitative study. *Arch Dis Child* 2006;91(6):491-4.

Confidential: For Review Only

24. McCormack LA, McBride CM, Paasche-Orlow MK. Shifting Away from a Deficit Model of Health Literacy. *Journal of Health Communication* 2016;21(sup2):4-5.

25. Dixon A, Le Grand J, Henderson J*, et al.* Is the British National Health Service equitable? The evidence on socioeconomic differences in utilization. *J Health Serv Res Policy* 2007;12(2):104-9.

26. Alderman H, Headey DD. How Important is Parental Education for Child Nutrition? *World Development* 2017;94:448-64.

27. Strull WM, Lo B, Charles G. Do patients want to participate in medical decision making? *JAMA* 1984;252(21):2990-94.

28. Public Health England. Child vision screening resources consultation. Available: https://www.gov.uk/government/consultations/child-vision-screening-resources (Accessed 12/01/2018). 2017.

29. Fejzic J, Emmerton L, Tett SE. Towards concordance in healthcare: perspectives of general practitioners, complementary and alternative medicine practitioners and pharmacists in Australia. *J Clin Pharm Ther* 2010;35(3):309-21.

30. Coulter A, Entwistle V, Gilbert D. Sharing decisions with patients: is the information good enough? *BMJ* 1999;318(7179):318-22.

31. Heijnders M, Van Der Meij S. The fight against stigma: An overview of stigma-reduction strategies and interventions. *Psychol Health Med* 2006;11(3):353-63.

Confidential: For Review Only

Table 1. Topic Guide (based on the Health Belief Model)[12]

CONCEPT	DEFINITION	AREAS OF QUESTIONING
Perceived susceptibility	An individual's assessment of their chances of developing a condition	Experience of eye conditions encountered in children<br>Experience of eye tests and or treatment as a child/adult<br>Eye care advice received from parents/friends<br>Advice they would give their child
Perceived severity	An individual's opinion as to the seriousness of the condition	Importance of vision: worries, fears, attitudes<br>Attitudes to children wearing glasses<br>Attitudes to children wearing eye patches<br>Knowledge of impact of poor vision on employment, driving and other everyday activities
Perceived benefits	An individual's opinion as to whether a new behaviour is better than current behaviour.	Importance of good vision<br>Impact of poor vision in children<br>Impact of vision on a child's ability to learn
Perceived barriers	An individual's opinion as to what will prevent them from adopting a new behaviour	Costs and relative costs<br>Satisfaction with health services<br>Number of visits to optometrist or Hospital<br>Obstacles to attending<br>Eye clinic opportunities<br>Cosmetic impact
Self-Efficacy	Belief in one's own ability to perform an action	Capability of arranging appointments<br>Knowledge of children's eye tests<br>Capability of performing the treatment (ensuring your child wears glasses or eye patch every day to improve their vision)
Cues to Action	Factors that will prompt a person into changing behaviour	Family attitude to eye care<br>Attitude of school to eye care<br>Any associated cultural or religious practices in relation to children's or adult eye care

Confidential: For Review Only

Table 2. Description of participants and indicators of the key themes highlighted at parental interview constructed using the Health Belief Model framework.

Parent	Ethnicity	Attended & adhered	Attended & non-adherent	Non-attendance & non-adherent	Susceptibility	Severity	Benefit	Barrier	Self-efficacy‡	Cues to Action
Mother 1 (i)	South Asian			Yes				x	x	
Mother 2 (i)	South Asian		Yes					x	x	
Mother 3	South Asian	Yes				x	x			x
Mother 4	African		Yes			x		x	x	
Mother 5	South Asian		Yes					x		
Father 1	White British	Yes				x	x			
Mother 6	White British	Yes			x		x	x	x	x
Mother 7	South Asian		Yes					x	x	x
Father 2	South Asian		Yes							
Mother 8	South Asian		Yes					x	x	
Mother 9	White British		Yes							
Mother 10	White British			Yes				x	x	
Mother 11	White British			Yes					x	
Mother 12	South Asian		Yes		x			x	x	x
Mother 13†	South Asian									
Father 3	South Asian	Yes			x	x	x	x		x
Mother 14 (i)	South Asian		Yes					x		
Mother 15	South Asian		Yes		x			x	x	x
Mother 16	South Asian		Yes		x			x	x	
Mother 17	South Asian			Yes					x	

i = interpreter
† = mother 13 and father 3 are parents of the same child and were interviewed together, only the fathers discourse is reported.
Key themes:
Severity = reduced vision stated as important.
Susceptibility = parental belief child has or could have an eye problem.
Barrier = stigma, language, transport, difficulty arranging appointment, child's fear.
Benefit = parental belief child's vision and/or general ability has improved with wearing spectacles.
Self-efficacy‡ = parents reported lack of self-efficacy; lack of confidence in child's ability to perform test, conflicting advice, lack of information.
Cues to action = school support or family support

Confidential: For Review Only

Table 3. Examples of illustrative quotes within each of the six key constructs of Health Belief Model

CONSTRUCT	Parental quotes
Perceived susceptibility	• They'd given me a letter to take to the opticians, but I didn't ever think nought from it, I just thought I dunno… Yeah but it's not my fault that like hers are like that, obviously she's born with eyes like that. (mother 11)<br>• […] has never been to an optician's appointment and she's never experienced any in school…. They've done it (eye test) in school but I was not part of it, I wasn't there. (mother 1)<br>• …they said to go and have him checked up but he's been scared. (mother 17)<br>• ….oh no she's too young so I thought she's too young and (erm) why she's going to wear glasses. (mother 12)
Perceived severity	• ….she's fine at home she doesn't need the glasses at home. She goes to the mosque as well and she will be doing reading there as well and she doesn't need them at mosque. She's asked the teacher there as well and she says she's fine and she doesn't need them there as well (mother 2).<br>• ….my wife was saying "she can see alright, she doesn't need to wear glasses." (father 2)<br>• …. he's got very short attention span so he wasn't interested so he just liked to look around so I don't know giving up and the like. So I was a bit concerned whether the eyesight was actually you know was a problem or not in that sense (mother 8).<br>• To be honest yesterday she took her glasses out of school and she thought oh I'm a very different girl, my friends are saying you're a different […] to be honest she doesn't need really, she's OK without glasses (mother 7).<br>• ….if a kid cannot see properly and keeps getting headaches because you know he's trying to concentrate, it's going to affect him in his education, it's going to affect him when he's playing and during life as well, so it's very important. (father 3)
Perceived benefits	• …. if he takes them off then his vision is going to get really weak. Glasses will help him so he has to keep them on all the time and just take them off when he really has to. (mother 3)<br>• Yeah because I always tell my children to wear glasses, especially when they are studying and watching TV and I try to encourage them because they do need (erm) you know when they are studying or watching tv. (mother 12)
Perceived barriers	• Awkward, really awkward because it's generally in school time and she doesn't like missing any time at school and it's awkward for me also working in school …. (mother 6).<br>• So (erm), many times many occasions I've forgot glasses at home and then I gave one pair to school as well to resolve this issue. (mother 12)<br>• If I knew that she had one of those wavering eyes then I would definitely have taken her but I didn't understand that from the letter. (mother 14)<br>• …but she didn't want the stigma of being bullied and being called pirate and things like that so I said she could wear them at home instead. (mother 6)<br>• …in Zimbabwe people who wear glasses they are seen as people who are really well educated. They say, "You're wearing glasses, you think you're so.. educated." (mother 4)
Self-Efficacy	• It would be good to be updated on whether her eyesight was getting better or worse (mother 2).<br>• ….they need to give us a report as well, comparing from the start to now (mother 16).
Cues to Action	• So it's just that everyone nearly in my family wears glasses so I'm happy for my son to wear it once his eyes have improved that's all (mother 3).<br>• …teachers were really supportive as well and actually they told me you can bring (the glasses)to school and we will help her to wear her glasses at school as well.(mother 12)

Confidential: For Review Only

BMJ Paediatrics Open

Confidential: For Review Only

A qualitative study investigating the perceptions of parents of children who failed vision screening at the age of 4-5 years.

Journal:	*BMJ Paediatrics Open*
Manuscript ID	bmjpo-2018-000307.R1
Article Type:	Original article
Date Submitted by the Author:	12-Jul-2018
Complete List of Authors:	Bruce, Alison; Bradford Institute for Health Research, ; University of York, Health Sciences<br>Sanders, Tom; University of Sheffield, School of Health and Related Research (ScHARR)<br>Sheldon, Trevor; University of York, Department of Health Sciences
Keywords:	Comm Child Health, Ophthalmology, Patient perspective, Qualitative research, Screening

SCHOLARONE™
Manuscripts

A qualitative study investigating the perceptions of parents of children who failed vision screening at the age of 4-5 years.

Alison Bruce (corresponding author)

Bradford Institute for Health Research, Bradford Teaching Hospitals NHS Trust, Duckworth Lane, Bradford, BD9 6RJ

01274 383414

alison.bruce@bthft.nhs.uk

Tom Sanders

School of Health and Related Research (ScHARR), Section of Public Health, University of Sheffield, Regent Court, 30 Regent Street, Sheffield, S1 4DA

0114 222 4293

tom.sanders@sheffield.ac.uk

Trevor A Sheldon

Department of Health Sciences, University of York, Heslington, York, YO10 5DD

01904 321521

Trevor.Sheldon@york.ac.uk

Key words: adherence, vision screening, spectacles, candidacy, children's eye services

Word count: 3472

Confidential: For Review Only

Abstract

Objective: To explore in depth parents' experiences and understanding of their children's eye care in order to better comprehend why there is relatively low uptake of services and variable adherence to treatment.

Design: Semi-structured interviews, informed by the Health Belief framework, were conducted with parents of children who had failed vision screening at age 4-5 years. Four were parents of children who never attended follow-up, 11 had children who attended but did not adhere to spectacle wear and 5 parents of children who had attended and adhered. Interviews were recorded and transcribed verbatim, thematic analysis based on the constant comparative method was undertaken.

Results: Parents beliefs led to uncertainty about the benefit of treatment, with parents testing their children to confirm the presence of a vision deficit and seeking advice from other family and community members. The stigma of spectacle wear explained the resistance of some to their child's treatment with the maintenance of 'normality' often more important than clinical advice. The combination of parents own health beliefs, stigma and the practicalities of attending appointments together influenced parental decisions. Attendance following vision screening and the decision to adhere to spectacle wear were primarily based on the perceived severity of the visual reduction with the perceived benefit of spectacle wear outweighing any negative consequences.

Conclusions: Health care professionals require a greater understanding of parents' decision making processes in order to provide personalised information. Knowledge of the cues to attendance and adherence provides policy makers a framework with which to review the barriers, develop strategies and redesign children's eye care pathways.

Confidential: For Review Only

INTRODUCTION

Decreased vision in young children is commonly due to the presence of refractive error, strabismus, and/or amblyopia.[1] The presence of one or more of these conditions can result in a visual deficit in the developing infant. Most young children with refractive error or amblyopia do not however demonstrate obvious signs or symptoms, making it difficult for parents to identify, and vision screening is recommended in the United Kingdom at age 4-5 years.[2] Early detection programmes can only be effective if those identified with poor vision are appropriately treated. In deprived communities both locally[3] and internationally[4] attendance rates following vision screening are reported between 30% to 40%.3 In addition studies have shown poor adherence to spectacle wear,[5-7] therefore a high proportion of children with identified needs do not access ophthalmic services and subsequent treatment. Socioeconomic factors have been reported to influence access to children's eye care[8] and adherence with spectacle wear.[9] In USA difficulty navigating the health care system, cost and loss and breakage of spectacles has been found to prevent attendance and adherence leading to inequalities in visual health.[10] Factors such as cultural sensitivity and health beliefs have been associated with differences in access, uptake and adherence decisions in adults.[11] People's perception of the risk of health problems also influences healthcare utilisation.[12] Current literature therefore provides evidence of a number of factors impacting on access and uptake of children's eye services. However, unlike adult ophthalmic services[13] where patients' experiences have been documented there is little reported qualitative evidence which would provide an understanding of parents' experiences of their child's eye care. This study explores in depth parents' experiences and understanding of their children's eye care in order to better comprehend why there is relatively low uptake of services and variable adherence to treatment.

Confidential: For Review Only

METHODS

Participant recruitment

The population based vision screening programme in Bradford, a large multi-ethnic city in England, is offered to children in their year of school entry, aged 4-5 years. Children failing to achieve the pass criterion set by the UK National Screening Committee[2] (≤0.20 logMAR in both eyes) are referred for examination, either to a community optometrist or the hospital eye service (HES) and subsequently followed-up in order to measure the VA with prescribed spectacles. As part of a separate longitudinal research programme[14] children were followed-up between 2013 and 2015 to explore the impact of adherence to spectacle wear on VA and early literacy. Parents of children participating in the study were invited for interview between November 2015 and May 2016. Parents were purposively selected if their child had failed vision screening in school years 2013-2014 or 2014-2015 and their child was either a non-attender (never attended follow-up appointments at their local optometrist or the Hospital Eye Service (HES) (non-attenders) or their child was non-adherent (not wearing prescribed spectacles at unannounced visits in school and/or spectacle wear was not corroborated by documentation in the medical notes). For comparison purposes, parents of four children identified as adherent (wearing prescribed spectacles at unannounced visits in school) were also invited to participate.

Initially forty letters with pre-paid reply slips were sent inviting parents (mother or father) of children participating in the longitudinal study to participate. No responses were obtained with this method. The letters were then distributed to the parents via their child's school. All interviews were undertaken in the school that their child was attending. Bradford is ranked the 5th highest deprived city in the UK, the five schools located across Bradford all had a Townsend score between 1 and 4 indicating lower socioeconomic status of the school communities. Four of the parents required interpreters (3 Urdu and 1 Parsi) the interpreters were teaching assistants working in the schools.

Confidential: For Review Only

The study was informed by the Health Belief Model (HBM), a theoretical concept developed in the arena of Public Health (Figure 1).[15] It consists of six key constructs which together provide an explanatory framework for the adoption of preventative health behaviour.[16] HBM was developed from the study of the factors influencing adults attendance at health screening programmes[15] so was chosen as a starting point to inform the topic guide for this study (Table 1). The topic guide was used to ensure details of the parents' experiences and understanding of their child's eye care was captured, in particular their views on vision and spectacle wear in children and the influence of their own and the experiences of family members (Table 1).

Semi-structured interviews were conducted by one member of the study team (AB), a female orthoptist and post-doctoral research fellow, who was unknown to the parents, in the child's school at a time suitable for the parent, two mothers in one school arrived at the same time and a combined interview was undertaken. Both the mother and father of one child were interviewed together; the father was the main contributor therefore only his discourse is included in the analysis. Interviews were audio recorded and transcribed verbatim, in addition, the non-English transcripts were listened to by a bilingual research assistant to confirm accuracy of the interpretation.

Two members of the study team (AB and TS) reviewed the transcripts independently and then together developed a thematic analysis, using constant comparison between the transcripts, identifying key themes and concepts.[17,18] Our approach to analysis was based on the 'constant comparative 'methodology derived from grounded theory. We analysed the interviews in search of similarities and differences which led to the development of themes in the data. Thematic analysis was chosen to allow for the emergence of opinions that were not anticipated in advance. No member checking was performed with this hard-to-recruit group of parents; however a focus group with a small number of mothers confirmed the interpretation of the findings. Written consent was obtained prior to each interview. Ethics approval was obtained from the National Research Ethics Committee Yorkshire and the Humber- South Yorkshire UK (Ref 13/YH/0379).

Confidential: For Review Only

RESULTS

In-depth interviews were completed with 20 parents (17 mothers and 3 fathers) of 19 children from five schools; 14 parents were South Asian, 5 were white British and 1 African heritage (Table 2). No further interviews were undertaken after saturation of the emerging themes from the 19 interviews. Our analysis identified themes which highlighted pragmatic reasons for non-attendance and non-adherence and also those related to health beliefs about illness, disease and consultation behaviour, illustrative quotes are presented in the text. The themes identified present the complexity of parental decision making.

Health Beliefs

Deep rooted health beliefs played an important part in explaining attitudes towards vision care in children starting primary school. Doubt along with other cultural health beliefs gave rise to uncertainty surrounding the potential benefits of spectacle wear, viewed by some as unnecessary.

Confidence in the test

Reduced vision in young children is not generally associated with overt signs and symptoms and parents found it difficult to accept their child had a vision problem. Adherence to spectacle wear was supported by parents when they perceived the vison test to be reliable.

> **It was really hard when she was young and it was the first time she had her eyes tested and they were putting some things in front of her eyes and I think she found that difficult. (mother 3, adherent)**

> **You do find the children do get bored very, very quickly but they dealt with them in a pleasant manner but sometimes it makes me wonder whether they actually could tell what she was seeing properly because, with them being so young. (mother 6, adherent)**

Confidential: For Review Only

He's never had any problems with his work to say that he's got bad eyes (mother 10, non-attender)

As he's got a bit older and he's obviously done more eye tests ….I'm a lot happier that (you know) he is properly diagnosed. (mother 8, non-adherent)

The view that children 'should not' require spectacles and the lack of confidence in the accuracy of testing young children led to parents testing their own children at home and canvasing significant others to verify that the child's vision was good without spectacles.

Mums tried her at home to check & test to see if she needs them but there is no difference so it's not like she needs them at home at all. (mother 3, adherent)

She does gymnastics as well and she doesn't wear her glasses and she can see on the beams and the floor and everything fine. (mother 6, adherent)

….she's fine at home she doesn't need the glasses at home. She goes to the mosque as well and she will be doing reading there as well and she doesn't need them at mosque. She's asked the teacher there as well and she says she's fine and she doesn't need them there as well (mother 2, non-adherent).

I says to her can you see without them and she says yeh I can see without them, yeh…she's had no problems at all, she can see alright, fine. She's had no pain in her eyes or anything like that, she says she doesn't really need them she can see everything without them (father 2, non-adherent)

Confidential: For Review Only

Community and social influences

The advice of family or community members was influential, particularly when it matched parents' personal beliefs. Where there was a positive family history of wearing spectacles parents reported attending and adherence with spectacle wear.

> **Well you do have more pressure from older people like my parents, "why is he wearing glasses, his eye sight is going to get worse, he'll get dependant on them." (mother 8, non-adherent).**

> **My mum has said it, like they're too young... I do have this from all my family, …. he is obviously too young and once he wears them they're going to be like you're going to have to wear them all the time. (mother 17, non-attender)**

> **He (father) doesn't feel that there is a problem so that's the reason why he hasn't taken her (child). (mother 1, non-attender).**

> **No one has discouraged the children from wearing glasses, everybody has asked … does she need glasses is that why she's wearing them…. so she gets a lot of encouragement. (mother 3, adherent)**

> **She was quite happy actually, because she's wearing them (glasses) like her older sister and brother and daddy…. (mother 12, non-adherent)**

Understanding

Parents of those children who were adhering to spectacle wear believed that failure to wear the spectacles would lead to a deterioration of both the child's vision and their ability to learn and many parents believed that by wearing the spectacles the vison would improve and then spectacles would no longer be required.

https://mc.manuscriptcentral.com/bmjpo

Confidential: For Review Only

…. if he takes them off then his vision is going to get really weak. Glasses will help him so he has to keep them on all the time and just take them off when he really has to. (mother 3, adherent)

…if a kid cannot see properly and keeps getting headaches because you know he's trying to concentrate, it's going to affect him in his education, it's going to affect him when he's playing and during life as well, so it's very important I think. (father 3, adherent)

…if she keeps wearing them now that when she gets to a teenager she might not need them (mother 6, adherent)

Stigma

The stigma of spectacle wear was reported by parents of adherent and non-adherent children and this contributed significantly in explaining parents' resistance to the child's spectacle wear.

To be honest yesterday she took her glasses out of school and she thought oh I'm a very different girl, my friends are saying you're a different […] to be honest she doesn't need really, she's OK without glasses (mother 5, non-adherent).

…in Zimbabwe people who wear glasses they are seen as people who are really well educated. They say, "You're wearing glasses, you think you're sooo… educated." (mother 4, non-adherent)

…because they're teasing him he's retaliated and we've had a few issues (father 3)

Confidential: For Review Only

Pragmatism

Access to appointments was influenced by the parent's ability to organise appointment times around busy working schedules, the children's school attendance and the availability of transport.

> **Awkward, really awkward because it's generally in school time and she doesn't like missing any time at school…. (mother 6, adherent).**

> **When I was at work it was difficult obviously because you know when you're at work you can't really get the appointments during the day and all the kids are at school..(mother 8, non-adherent)**

> **No, it's just main problem is parking, I just missed two appointments because I couldn't get the parking on time (mother 7, non-adherent)**

Information

The amount and detail of information provided to parents varied and parents suggested that more information was always helpful. Two mothers suggested that the eye care professional should provide parents with an indication of the improvement in VA at each visit.

> **We've always had leaflets given to us every time we've been so we were given plenty of information. (mother 6, adherent)**

> **I wasn't fully clear of the letter and what information I did take from it. (mother 1, non-attender).**

Confidential: For Review Only

They (eye professionals) need to understand a little better and give a percentage, so we (parents) can compare vision to the last test. (mother 16, non-adherent).

It would be good to be updated on whether her eyesight was getting better or worse (mother 2, non-adherent)

Parents whose first language was not English did not always fully comprehend the information provided or relied on family members to access care and interpret clinical advice.

…my older daughters can read now so that they can read and interpret letters (mother 3, adherent).

'I used to take my sister in law, who can understand English, so she can explain it and ask questions. (mother 14, non-adherent).

Supporting Strategies

Schools played a key role in supporting the parents in developing strategies to both attend and to ensure spectacle wear with some parents negotiating appropriate absence times for their child during school hours.

They were fine, the times that they gave me have always been in the afternoon instead of the morning so it's easy…and I can take them out of school (mother 2, non-adherent).

So (erm), many times many occasions I've forgot glasses at home and then I gave one pair to school as well to resolve this issue (mother 12, non-adherent).

Confidential: For Review Only

My son needs [glasses] all the time, so I have to say all the time to class teacher "please remind him to wear his glasses." (mother 16, non-adherent).

DISCUSSION

The decision to attend following vision screening and to adhere to spectacle wear was primarily based on the perceived severity of the visual reduction with the benefit of spectacle wear outweighing any negative consequences. Parental observations played an important role in validating the professional assessment, when parental perception did not match clinical opinion adherence was less likely.[19] Parental knowledge tended to dominate decisions where there was discordance between professional and lay knowledge[20] with parents having difficulty accepting clinical advice, particularly when their child did not appear to require spectacles for reading and writing. It has been reported that limited experience of a health condition affects judgement of its severity[21] this may also have influenced the parents reasoning to support spectacle wear in their children. Parents whose children had failed vision screening and had not taken their child for further assessment were keen to justify their actions by normalising the eye condition[19,22] in addition they did not report any support from either family members or from their child's school, this lack of support may have contributed to non-attendance. Stigma played a major part in explaining parents' resistance to clinical advice[23] Appearing 'normal' seemed to outweigh potential benefits[24] and was a strong driver for maintaining a socially acceptable image of the family within the community;[22] a child with spectacles seemed to threaten this image suggesting that the eye condition was not the sole factor impacting on adherence.[25] Parents who did not consider their child's vision to be impaired, who described barriers such as organising transport or social stigma did not follow recommendations and deemed treatment unnecessary. Parents with limited English language ability relied on family members to access care and interpret clinical advice, this use of the family network is reported to be unreliable, leading to misinformation and has been reported to impact on the child's care.[12] The practical

Confidential: For Review Only

considerations of attending regular appointments particularly in cases of uncertainty about the benefit of treatment therefore posed a dilemma, contributing to and perhaps being the tipping point for non-attendance and non-adherence. Where support was available either from family members or from the child's school this provided a cue to action prompting parents to take the first step in attending appointments.

Strengths and Limitations

This qualitative study helps to explain the reasons for non-attendance and poor adherence to spectacle wear in young children following vision screening. The strengths of the study include the purposive sample from a multi-ethnic community, including parents whose children were known to fail to attend and those whose children failed to adhere to prescribed spectacle wear, these are hard-to-reach groups of parents. The additional inclusion of parents whose children had attended and adhered to spectacle wear allowed insights into both barriers and enablers to attendance and adherence.

The children were participants in the BiB cohort and this could have encouraged positive responses in recruitment and in the views provided, however, no parent responded solely from the initial invitation to participate generated via BiB. The schools were crucial to the successful recruitment of parents and this may have positively influenced the parental responses regarding the support provided by schools, however all the parents declined to be interviewed in their own home and would only be interviewed in school. The participating parents were all recruited from deprived communities and this study may not be representative of the experiences of parents in more affluent areas, however non-attendance and non-adherence is known to occur predominantly in areas of lower socio-economic status[26] and therefore these findings will represent the experiences of this under reported, hard-to-reach group of parents.

https://mc.manuscriptcentral.com/bmjpo
Confidential: For Review Only

Existing Literature

The findings from this study provide a comprehensive picture of the experiences of parents whose young children have been referred following vision screening, further explaining the reasons for non-attendance and non-adherence. Previous population based studies reporting children's adherence to spectacle wear have focused on the factors found to be associated with non-attendance, reporting higher proportions of children from families of lower socio-economic status[26] failing to attend and older children demonstrating a greater rate of non-adherence to spectacle wear.[27] In a study of young children (under eight years) in Berkshire[28] compliance with spectacle wear was reported to be good, however, the study was undertaken is in a relatively affluent area and the parents were regular attenders to the hospital eye service.

Similar to our findings, studies of the experiences of adults in screening programmes describe that a lack of perceived severity of the condition influences attendance.[13,29] This study, in the field of children's eye care, adds to this, reporting that where the severity of the condition is in doubt the parent will perform their own confirmatory vision test in their home environment. The practical barriers to attendance we report such as access to appointments and transport also confirm the findings of previous studies.[13,29] In addition, importantly, this study highlights the influence that support from significant others such as family or school have in helping overcome barriers to attendance and adherence.

Implications for clinicians and policy makers

Pragmatic strategies are required to improve attendance and adherence. The desire for condition specific information is underestimated by clinicians[30] and provision of personalised information, following vision screening, highlighting the benefits of treatment in young children could improve attendance. The current national development of information leaflets specifically aimed at parents providing information following vision screening may influence attendance.[31] Concordance around treatment should involve discussion between clinician and parent through which an informed decision regarding treatment can take place.[32] This

Confidential: For Review Only

has been reported in general practice with patients less likely to take medications if their own concerns are not initially addressed.[20] Further study is required into how eye care professionals communicate with parents, and how they present information[33] to ensure the parents can make informed decisions regarding adherence. Stigma was commonly cited by parents as a barrier to adherence.[34] Strategies both at the individual and community level are required with the role of schools as facilitators in reducing stigma and promoting adherence, an area highlighted by parents and requiring future study.

These findings provide insight into the reasons parents either rejected or resisted therapeutic advice, not solely reflecting levels of knowledge, but reflecting an active evaluation of the potential severity of the vision loss and the perceived loss of normality and social stigma compared to the benefit of the treatment. Our results illustrate the complexity around attendance and adherence patterns and provide a greater understanding which can inform the redesign of children's eye care pathways.

Acknowledgments: We thank all the families and schools who took part in this study.

Competing interests: None declared

Grants: AB is funded by a National Institute for Health Research Post-Doctoral Fellowship Award (PDF-2013-06-050). The views expressed are those of the author(s) and not necessarily those of the NHS, the NIHR or the Department of Health.

Contributor statement: AB initiated the project, designed the study, conducted the interviews, analysed the transcripts, drafted and revised the paper. She is guarantor. TS analysed the transcripts and revised the draft paper. TAS initiated the project and revised the draft paper.

Confidential: For Review Only

"What is already known on this topic"

- Reduced vision in young children is commonly related to refractive error, strabismus or amblyopia.
- Following identification of reduced vision at vision screening attendance for eye care appointments is poor with 30% of children failing to attend.
- Treatment consists of spectacle wear and may be combined with wearing an eye patch but adherence to treatment is inconsistent.

"What this study adds"

- Reasons for non-attendance and non-adherence are complex with both pragmatic factors and health beliefs interlinked in parental decision making.
- The decision to adhere to spectacle wear was based primarily on the perceived severity of the visual reduction with the perceived benefit outweighing any negative consequences.
- The role of schools as facilitators should be considered when developing interventions to promote spectacle wear in young children.

Figure Legend

Figure 1 Illustration of Health Belief Model

Confidential: For Review Only

REFERENCES

1. Robaei D, Rose K, Ojaimi E*, et al.* Visual Acuity and the Causes of Visual Loss in a Population-Based Sample of 6-Year-Old Australian Children. *Ophthalmology* 2005;112(7):1275-82.

2. National Screening Committee. The UK NSC policy on Vision defects screening in children 2013. Available: http://www.screening.nhs.uk/vision-child (Accessed 11/12/2017).

3. Bruce A, Outhwaite L. Uptake, referral and attendance: results from an inner city school based vision screening programme. *Br Ir Orthopt J* 2013;10:41 - 45.

4. Slingsby TJ, Mallory P, Spencer T. Childhood Vision Screening in Western South Dakota: Examining Barriers to Post-Vision Screening Follow-up Referral. *S D Med* 2017;70(1):25-31.

5. Sharma A, Congdon N, Patel M*, et al.* School-based Approaches to the Correction of Refractive Error in Children. *Survey of Ophthalmology* 2012;57(3):272-83.

6. Aldebasi YH. A descriptive study on compliance of spectacle-wear in children of primary schools at Qassim Province, Saudi Arabia. *Int J Health Sci (Qassim)* 2013;7(3):291-99.

7. Maconachie GD, Farooq S, Bush G*, et al.* Association Between Adherence to Glasses Wearing During Amblyopia Treatment and Improvement in Visual Acuity. *JAMA ophthalmology* 2016;134(12):1347-53.

8. Williams C, Northstone K, Howard M*, et al.* Prevalence and risk factors for common vision problems in children: data from the ALSPAC study. *Br J Ophthalmol* 2008;92(7):959-64.

9. Ma X, Zhou Z, Yi H*, et al.* Effect of providing free glasses on children's educational outcomes in China: cluster randomized controlled trial. *BMJ* 2014;349.

10. Messer DH, Mitchell GL, Twelker JD*, et al.* Spectacle wear in children given spectacles through a school-based program. *Optometry and vision science : official publication of the American Academy of Optometry* 2012;89(1):19-26.

11. King's Fund Briefing *Access to health care and minority ethnic groups.* Februay 2006. Available: https://www.kingsfund.org.uk/sites/default/files/field/field_publication_file/access-to-health-care-minority-ethnic-groups-briefing-kings-fund-february-2006.pdf (accessed 20/12/2017).

Confidential: For Review Only

12. Dixon-Woods M, Kirk M, Agarwal S*, et al.* Vulnerable Groups and Access to Health Care:A Critical Interpretive Review.: National Co-ordinating Centre for NHS Service Delivery and Organisation London: 2005. Available online at http://www.menshealthforum.org.uk/uploaded_files/SDOvulnerablegroups2005.pdf [Accessed 09 November 2017].

13. Hipwell AE, Sturt J, Lindenmeyer A*, et al.* Attitudes, access and anguish: a qualitative interview study of staff and patients' experiences of diabetic retinopathy screening. *BMJ open* 2014;4(12):e005498.

14. Bruce A, Kelly B, Chambers B*, et al.* The effect of adherence to spectacle wear on early developing literacy: a longitudinal study based in a large multiethnic city, Bradford, UK. *BMJ open* 2018;8(6):e021277.

15. Rosenstock IM, Strecher VJ, Becker MH. Social learning theory and the Health Belief Model. *Health Educ Q* 1988;15(2):175-83.

16. Griffin MJ. Health belief model, social support, and intention to screen for colorectal cancer in older African American men. . *Health Promotion & Education* 2012.;51(1):12-22.

17. Glaser G, Stauss, A. *The discovery of grounded theory. Chicago: Aldine,*, 1967.

18. Pope C, Ziebland S, Mays N. Analysing qualitative data. *BMJ* 2000;320(7227):114-16.

19. McLachlan S, Mansell G, Sanders T*, et al.* Symptom perceptions and help-seeking behaviour prior to lung and colorectal cancer diagnoses: a qualitative study. *Fam Pract* 2015;32(5):568-77.

20. Britten N, Stevenson FA, Barry CA*, et al.* Misunderstandings in prescribing decisions in general practice: qualitative study. *BMJ* 2000;320(7233):484-88.

21. Bond L, Nolan T. Making sense of perceptions of risk of diseases and vaccinations: a qualitative study combining models of health beliefs, decision-making and risk perception. *BMC public health* 2011;11(1):943.

22. Prout A, Hayes L, Gelder L. Medicines and the maintenance of ordinariness in the household management of childhood asthma. *Sociol Health & Illn* 1999;21(2):137-62.

Confidential: For Review Only

23. Goffman E. *Stigma: Notes on the Management of Spoiled Identity*. Englewood Cliffs, N.J: Prentice-Hall, Inc, 1963.

24. Dixon-Woods M, Awan M, Gottlob I. Why is compliance with occlusion therapy for amblyopia so hard? A qualitative study. *Arch Dis Child* 2006;91(6):491-4.

25. McCormack LA, McBride CM, Paasche-Orlow MK. Shifting Away from a Deficit Model of Health Literacy. *Journal of Health Communication* 2016;21(sup2):4-5.

26. O'Colmain U, Low L, Gilmour C*, et al.* Vision screening in children: a retrospective study of social and demographic factors with regards to visual outcomes. *BJO* 2016;100(8):1109-13.

27. Holguin AMC, Congdon N, Patel N*, et al.* Factors Associated with Spectacle-Wear Compliance in School-Aged Mexican Children. *IOVS* 2006;47(3):925-28.

28. Horwood AM. Compliance with first time spectacle wear in children under eight years of age. *Eye* 1998;12:173.

29. Eborall H, Davies R, Kinmonth AL*, et al.* Patients' experiences of screening for type 2 diabetes: prospective qualitative study embedded in the ADDITION (Cambridge) randomised controlled trial. *Bmj* 2007;335(7618):490.

30. Strull WM, Lo B, Charles G. Do patients want to participate in medical decision making? *Jama* 1984;252(21):2990-94.

31. Public Health England. Child vision screening resources consultation. Available: https://www.gov.uk/government/consultations/child-vision-screening-resources (Accessed 12/01/2018). 2017.

32. Jordan JL, Ellis SJ, Chambers R. Defining shared decision making and concordance: are they one and the same? *Postgrad Med J* 2002;78(921):383-84.

33. Coulter A, Entwistle V, Gilbert D. Sharing decisions with patients: is the information good enough? *BMJ : British Medical Journal* 1999;318(7179):318-22.

34. Heijnders M, Van Der Meij S. The fight against stigma: An overview of stigma-reduction strategies and interventions. *Psychol Health Med* 2006;11(3):353-63.

Confidential: For Review Only

Table 1. Topic Guide (based on the Health Belief Model[14])

CONCEPT	DEFINITION	AREAS OF QUESTIONING
Perceived susceptibility	An individual's assessment of their chances of developing a condition	Experience of eye conditions encountered in children<br>Experience of eye tests and or treatment as a child/adult<br>Eye care advice received from parents/friends<br>Advice they would give their child
Perceived severity	An individual's opinion as to the seriousness of the condition	Importance of vision: worries, fears, attitudes<br>Attitudes to children wearing glasses<br>Attitudes to children wearing eye patches<br>Knowledge of impact of poor vision on employment, driving and other everyday activities
Perceived benefits	An individual's opinion as to whether a new behaviour is better than current behaviour.	Importance of good vision<br>Impact of poor vision in children<br>Impact of vision on a child's ability to learn
Perceived barriers	An individual's opinion as to what will prevent them from adopting a new behaviour	Costs and relative costs<br>Satisfaction with health services<br>Number of visits to optometrist or Hospital<br>Obstacles to attending<br>Eye clinic opportunities<br>Cosmetic impact
Self-Efficacy	Belief in one's own ability to perform an action	Capability of arranging appointments<br>Knowledge of children's eye tests<br>Capability of performing the treatment (ensuring your child wears glasses or eye patch every day to improve their vision)
Cues to Action	Factors that will prompt a person into changing behaviour	Family attitude to eye care<br>Attitude of school to eye care<br>Any associated cultural or religious practices in relation to children's or adult eye care

Confidential: For Review Only

Table 2 Participant Characteristics.

Parent	Ethnicity	Attended & adherent	Attended & non-adherent	Non-attendance & non-adherent
Mother 1 (i)	South Asian			Yes
Mother 2 (i)	South Asian		Yes	
Mother 3	South Asian	Yes		
Mother 4	African		Yes	
Mother 5	South Asian		Yes	
Father 1	White British	Yes		
Mother 6	White British	Yes		
Mother 7	South Asian		Yes	
Father 2	South Asian		Yes	
Mother 8	South Asian		Yes	
Mother 9	White British		Yes	
Mother 10	White British			Yes
Mother 11	White British			Yes
Mother 12	South Asian		Yes	
Mother 13*	South Asian			
Father 3	South Asian	Yes		
Mother 14 (i)	South Asian		Yes	
Mother 15	South Asian		Yes	
Mother 16	South Asian		Yes	
Mother 17	South Asian			Yes

i = interpreter
* = mother13 and father 3 are parents of the same child and were interviewed together, only the fathers discourse is reported.

Confidential: For Review Only

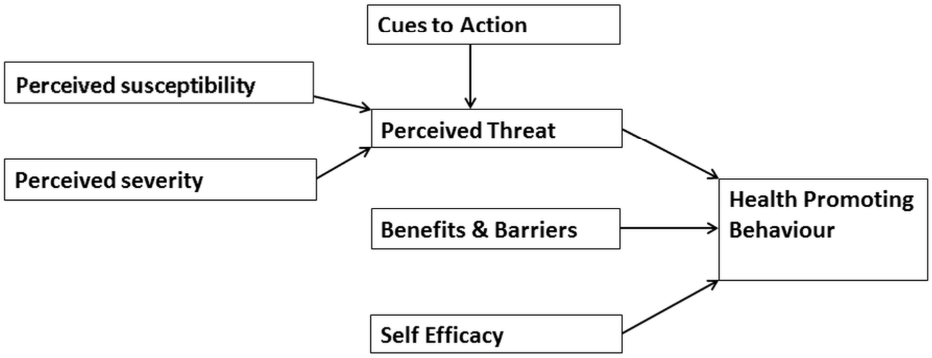

254x190mm (300 x 300 DPI)

review Only
